# Orientation and Membrane Partition Free Energy of PeT-Based Voltage-Sensitive Dyes from Molecular Simulations

**DOI:** 10.1021/acs.jpcb.3c08090

**Published:** 2024-03-09

**Authors:** Chun Kei Lam, Lap Yan Fung, Yi Wang

**Affiliations:** Department of Physics, The Chinese University of Hong Kong, Shatin, Hong Kong SAR, China

## Abstract

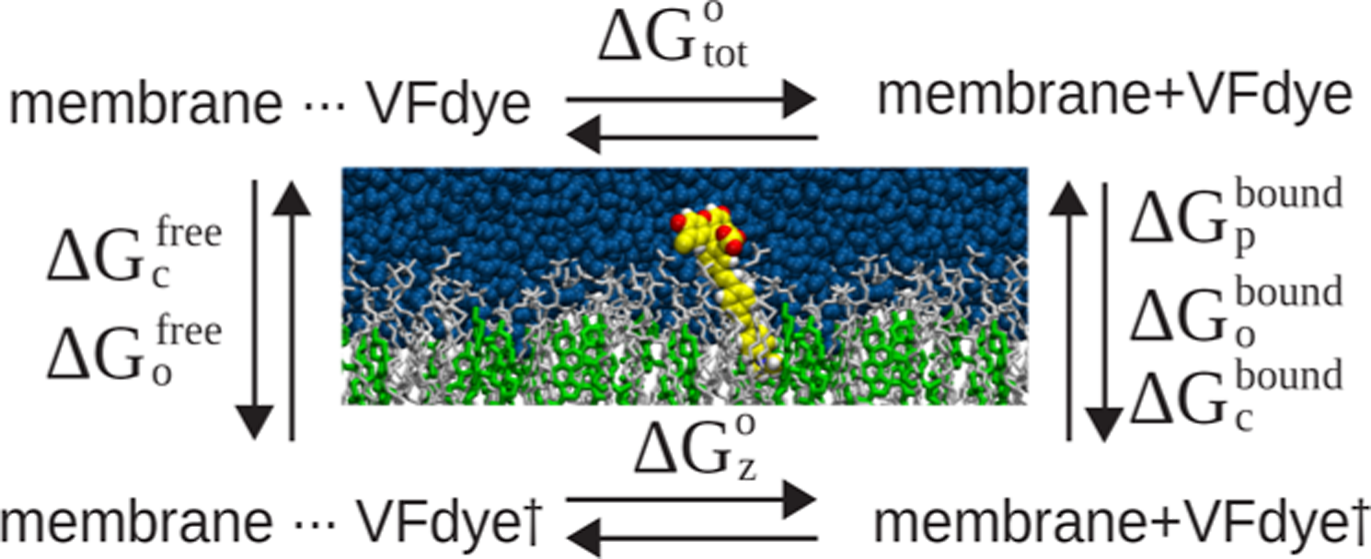

Voltage measurement via small-molecule fluorescent indicators is a valuable approach in deciphering complex dynamics in electrically excitable cells. However, our understanding of various physicochemical properties governing the performance of fluorescent voltage sensors based on the photoinduced electron transfer (PeT) mechanism remains incomplete. Here, through extensive molecular dynamics and free energy calculations, we systematically examine the orientation and membrane partition of three PeT-based voltage-sensing VoltageFluor (VF) dyes in different lipid environment. We show that the symmetry of the molecular scaffold and the net charge of the hydrophilic headgroup of a given VF dye dominate its orientation and membrane partition, respectively. Our work provides a mechanistic understanding of the physical properties contributing to the voltage sensitivity, signal-to-noise ratio, as well as membrane distribution of VF dyes and sheds light onto rational design principles of PeT-based fluorescent probes in general.

## Introduction

Understanding the patterns of neuronal signal transduction is essential in mapping the information-processing mechanism in the human brain. Such signal transduction routinely propagates through a change in the transmembrane potential of excitable cells, i.e., the action potential. While patch-clamp electrophysiology, which involves a direct insertion of electrodes into cells of interest, remains the standard approach to determine transmembrane potentials, its applicability is often limited by the high mechanical invasiveness and low throughput of the technique.^1^ Measurement of the transmembrane potential with voltage-sensitive fluorescent probes provides an attractive alternative to circumvent the above disadvantages.^2−4^ A large set of these probes, such as the VoltageFluor (VF) dyes,^5−8^ operate via a photoinduced electron transfer (PeT) mechanism and have found applications in a wide range of biological systems.^9−11^

Each VF dye consists of a xanthene-based fluorophore that acts as an electron acceptor and an aniline-based electron donor linked together by a phenylenevinylene molecular wire.^12^ Once the fluorophore becomes photoexcited, electron transfer from the donor to the acceptor may occur, which directly competes with fluorescence emission from the excited acceptor. With the VF dye residing in the outer leaflet of the plasma membrane, the outcome of this competition becomes voltage dependent; at resting or hyperpolarizing potentials, the transmembrane electric field accelerates the electron transfer, thus, quenching the fluorescence, and at depolarizing potentials, the transmembrane electric field is reduced or even reversed in direction, which decelerates the electron transfer, resulting in a brighter fluorescence signal.^13^ By analogy to electrochromic dyes,^14,15^ the voltage sensitivity of a VF dye can be modeled by the following simplified equation^12,16^
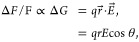
1where Δ*F*/*F* is the fractional voltage sensitivity of the VF dye, Δ*G* is the free energy change during the electron transfer, *q* is the amount of charge transferred, while *E⃗* and *r⃗* are the electric field vector and the displacement vector of the transferred electron, respectively (*r* and *E* represent their magnitudes), and θ is the angle between the two vectors. The linear dependence of their voltage sensitivity on the transmembrane potential implied by eq 1 has indeed been observed for VF dyes over the physiological range of ±100 mV.^12^

For a given VF dye, its maximum voltage sensitivity Δ*F*/*F* is achieved when θ = 0°, at which orientation its molecular wire is parallel to the membrane normal and the distance traveled by the electron reaches the maximum value of *r*. If the molecular wire is perpendicular to the membrane normal, i.e., θ = 90°, the electron will move perpendicularly to the electric field, and the dye will not respond to any variation in the transmembrane potential. All else being equal, the relative voltage sensitivities of two VF dyes can thus be estimated by comparing their tilt angles in a lipid membrane. Apart from orientation, the distribution of VF dyes over various cellular membranes and water is another important consideration in their rational design. In principle, a greater affinity of a VF dye toward the membrane (relative to water) should result in a larger signal-to-noise ratio in the fluorescence measurement. Moreover, a recently developed PeT-based probe^17^ has been found to reside primarily in the mitochondrial membrane, further attesting the relevance of understanding the partition of these fluorescent voltage sensors.

Previously, our group employed MD simulations to examine VF dye orientation within a POPC membrane, which led to the development of disulfoVF2.1.Cl, or dsVF in short, that has significantly higher voltage sensitivity than its predecessor monosulfoVF2.1.Cl, or msVF, due to a better alignment between the former VF dye and the membrane normal.^16^ Despite the success of dsVF, our understanding of different physicochemical properties that may govern the orientation and distribution of VF dyes within various lipid environment remains limited. In this work, we perform a systematic comparison of three members of the VF dye family, namely, msVF, dsVF, and the newly designed isoVF,^18^ based on their orientation profiles obtained from plain MD simulations in a pure POPC, a POPC/cholesterol mixed bilayer, as well as a mammalian membrane. In addition, we determine the partition free energy of these fluorescent probes toward a lipid bilayer via the adaptive biasing force (ABF) approach^19−22^ and a simulation strategy originally developed for protein–ligand binding.^23−25^ Our calculations reveal that the location of the sulfonate group(s) and the overall symmetry of its structure dominate the orientation of a VF dye in the membrane, with lipid composition also playing an important part. The membrane affinity of a VF dye has a strong dependence on the dye’s net charge, which is the key physical property that distinguishes its membrane partition from its peers. Based on these findings, we discuss considerations in the rational design of VF dyes aimed at improving their performance through modification of their molecular scaffolds.

## Methods

The orientation of a VF dye, measured by its tilt angle θ with respect to the membrane normal, can be readily extracted from plain MD simulation trajectories. However, computation of its partition free energy to a membrane, Δ*G*_tot_, is less straightforward. For clarity, we first define Δ*G*_tot_ below and then outline our protocols for its computation, with more details provided in the Supporting Information.

### Membrane Partition Free Energy of the VF Dye

**Figure 1:**
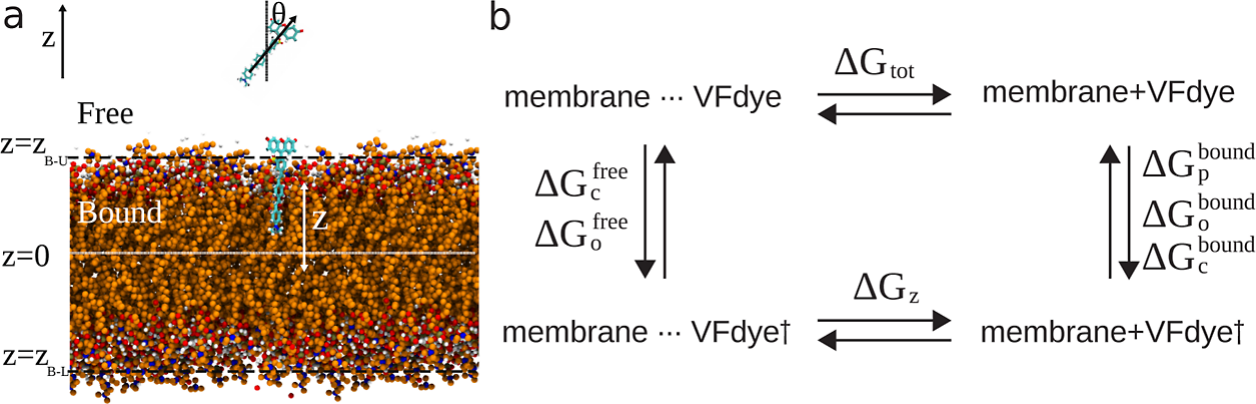
(a) Schematic illustration of a VF dye partitioning to a lipid membrane. θ is the tilt angle characterizing the orientation of the VF dye relative to the membrane normal. The *z* coordinate of the COM of the VF dye is measured relative to the average position of phosphorus atoms in the bilayer (*z* = 0). *z* = *z*_B-U_ and *z* = *z*_B-L_ together define the bound domain: the VF dye is considered to be bound, e.g., partitioned into the lipid phase, if *z*_B-L_ ≤ *z* ≤ *z*_B-U_ and otherwise free, e.g., in solution. (b) Thermodynamic cycle of determining the membrane partition free energy of a VF dye (Δ*G*_tot_) via the PMF approach.

Consider a system with a lipid bilayer of lateral surface area *A* placed in a water box (Figure 1) and assume that a total of *N* identical VF dyes are present, out of which *N*_b_ dyes are inserted into the membrane and *N*_f_ dyes are in the solution. Here, we consider the *N*_b_ dyes inserted (partitioned) into the membrane to be in the bound state, while the *N*_f_ dyes in the solution are in the free state. At equilibrium and in the limit of low VF dye concentration, Δ*G*_tot_, the membrane partition free energy of a VF dye, can be obtained from the ratio of the configurational partition functions of the dye, per unit volume, in the bound and the free states.^26−29^ Unlike in a homogeneous phase, VF dyes partitioned into a membrane do not distribute uniformly in the lipid bilayer and instead reside at the membrane–water interface with a preferred orientation (tilt angle). Such a preference in both location and orientation of its “bound” form suggests that Δ*G*_tot_ computation of a VF dye may benefit from strategies developed for efficient protein–ligand and protein–protein binding free energy calculations. Here, following the previous work,^23^ we first cast Δ*G*_tot_ as (see the Supporting Information for details)
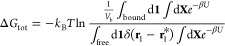
2where *U* is the potential energy of the system, **1** and **X** represent the degrees of freedom of an arbitrarily chosen VF dye molecule and the remaining atoms (other dyes, lipids, water, and ions), respectively, **r**_1_ is the position vector of the center of mass (COM) of the chosen VF dye, and **r**_1_* refers to any arbitrary point in the free solution. The subscripts “bound” and “free” indicate the relevant part of the configurational space (the lipid and the water phase, respectively) over which the integration is to be carried out. The volume of the bound domain is *V*_b_ = *A*(*z*_B-U_ – *z*_B-L_), where *z*_B-U_ and *z*_B-L_ represent the upper and lower boundaries of the membrane, respectively. The resulting Δ*G*_tot_, in analogy to the previously studied peptide–membrane insertion energy,^30^ can be interpreted as the free energy difference between (i) 1 mol of VF dyes in 1 L of the lipid membrane and (ii) 1 mol of VF dyes in 1 L of water.

In order to determine Δ*G*_tot_ efficiently, we adopt the simulation strategy by Woo and Roux^23^ and subsequently extended by Gumbart et al.^24,31−33^ that employs a series of restrained intermediate states. Briefly, a VF dye in the membrane-bound state is first restrained positionally, conformationally, and orientationally by applying a restraining potential on the *z* coordinate of its COM, the root-mean-square deviation (RMSD, denoted ξ hereon) in its atomic positions, and the tilt angle θ, respectively, and sequentially. The losses in positional, conformational, and orientational degrees of freedom are associated with the free energy change Δ*G*_p_^bound^, Δ*G*_c_^bound^, and Δ*G*_o_^bound^, respectively (Figure 1). The conformationally and orientationally restrained VF dye is then transferred reversibly from its restrained position to the free solution, with −Δ*G*_*z*_ representing the free energy change during this process. Finally, all restraints imposed on the VF dye are removed in the free solution, giving rise to −Δ*G*_o_^free^ and −Δ*G*_c_^free^. The membrane partition free energy Δ*G*_tot_ is recovered by summing up the individual contributions, with their signs determined by the thermodynamic cycle (Figure 1)
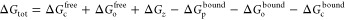
3

Out of the six components of Δ*G*_tot_, four (Δ*G*_c_^bound^, Δ*G*_o_^bound^, Δ*G*_*z*_, and Δ*G*_c_^free^) are determined from the potential of mean force (PMF) along the relevant transition coordinates, i.e., collective variables (CVs), while the remaining two are computed from plain MD simulations (Δ*G*_p_^bound^) or numerical integration (Δ*G*_o_^free^). Below, we further describe the computation of Δ*G*_p_^bound^ and Δ*G*_*z*_, leaving the rest of Δ*G*_tot_ components’ calculation to the Supporting Information. Note that compared to protein–ligand or protein–protein binding free energy calculations,^23,24^ where seven collective variables, namely, RMSD, three spherical coordinates, and three Euler angles, need to be employed, only three CVs (RMSD, θ and *z*) are required for membrane partition free energy calculations considered here.

### Δ*G*_p_^bound^ from Positional Restraint in the Bound State

We introduce a “flat-bottom” positional restraint *u*_p_ to bias a VF dye toward the most probable position it adopts in the membrane-bound state
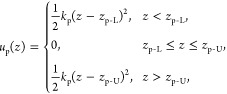
4where *z* represents the *z* coordinate of the COM of the VF dye measured from the bilayer center, *z*_p-L_ and *z*_p-U_ are the lower and upper boundaries, respectively, of a confined region within the membrane, and *k*_p_ is the force constant that quantifies the strength of the positional restraint. Note that *z*_p-L_ and *z*_p-U_ are not to be confused with *z*_B-L_ and *z*_B-U_, the latter defines the bound domain of a VF dye, which is determined by the phase boundaries of the lipid membrane, and the former defines the most probable or favored position of the VF dye within a membrane, which corresponds to a small part around the peak of its positional probability density distribution. The free energy cost Δ*G*_p_^bound^ due to *u*_p_ is obtained as
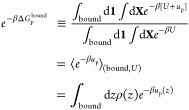
5where ρ(*z*) is the probability density distribution of the *z* coordinate of the COM of the VF dye. Δ*G*_p_^bound^ is then determined by numerically integrating the above equation using ρ(*z*) obtained from restraint-free, equilibrium MD simulations.

### Δ*G*_*z*_ as the Membrane Partition Free Energy of a Geometrically Restrained VF Dye

The membrane partition free energy Δ*G*_*z*_ of a positionally, conformationally, and orientationally restrained VF dye is obtained from
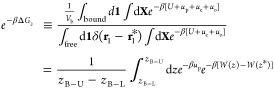
6where the PMF *W*(*z*) is determined by the extended-system adaptive biasing force (eABF)^34,35^ method with the corrected z-averaged restraint (CZAR) scheme in Colvars,^36^ in the presence of the conformational restraint *u*_c_ and orientational restraint *u*_o_. Derivation of the above equation is provided in the Supporting Information, while a similar expression (without the positional restraint term) for peptide–membrane binding can be found in the study by Vivcharuk et al.^28^ As described previously, *z*_B-U_ and *z*_B-L_ correspond to the boundaries of the lipid phase and *z*_B-U_ – *z*_B-L_ is set to the average membrane thickness measured for a given bilayer (38.6 for POPC and 44.7 Å for POPC/CHL, see Table S1).

### MD Simulation and Analysis Protocols

MD simulation systems with either an msVF, a dsVF, or an isoVF molecule were constructed by first inserting the dye vertically into a leaflet of a pure 1-palmitoyl-2-oleoyl-*sn*-glycero-3-phosphocholine (POPC) membrane containing 84 POPC lipids or a mixed membrane containing 76 POPC lipids and 38 cholesterol molecules [POPC/CHL (2:1)]. Both membranes were previously equilibrated in a 1 μs simulation^37^ performed on the specialized machine Anton.^38^ A lipid that overlapped with the VF dye was then deleted. Using the solvate and autoionize plugin of VMD,^39^ the systems were solvated with water and then neutralized by sodium and chloride ions at a concentration of 0.15 M. Final simulation systems contain approximately 28,000 atoms, with a size of approximately 52 × 52 × 100 Å^3^. In addition, each VF dye was inserted into a model mammalian plasma membrane, with parameters and equilibrated structures from our previous work,^40^ which had been constructed based on the coarse-grained simulations by Ingólfsson et al.^41^ The VF dye was inserted into the outer leaflet of this asymmetric mammalian membrane, corresponding to the location where it senses the transmembrane voltage change in experiments.^3^ This simulation system contained approximately 48,000 atoms with a size of approximately 78 × 78 × 75 Å^3^. Finally, to improve sampling, a two-dye system was constructed for the POPC/CHL bilayer, where one VF dye was inserted into each leaflet. This two-dye system was used to collect statistics in plain MD simulations, while all free energy calculations were conducted on single-dye systems.

An energy minimization of 5000 steps was first performed for each VF dye-membrane system, followed by three replicas of 500 ns equilibrium simulations, resulting in an aggregated simulation time of 13.5 μs for all systems. The CHARMM36 force field for lipids^42^ and small molecules^43,44^ was employed in all simulations. Parameterization of msVF and dsVF has been described in our previous work,^16^ while the parameters of isoVF were obtained from transposing of msVF functional groups. All simulations were performed with the NAMD package^45^ 2.13 release. Covalent bonds involving hydrogen atoms were constrained using RATTLE.^46^ The geometry of water was maintained using SETTLE.^47^ The multiple-time step algorithm, in which short-range and long-range interactions were calculated with a time step of 2 and 4 fs, respectively, was employed. The cutoff for short-range nonbonded interactions was set to 12 Å, with a switching distance of 10 Å. The CHARMM force switching was used for vdW forces in order to be consistent with the CHARMM36 force field for lipids. The long-range electrostatic interactions were modeled using the particle mesh Ewald (PME) method^48^ with a grid density of 1 Å^–3^. Langevin dynamics with a damping coefficient of 1 ps^–1^ was used to keep the temperature at 310 K, while a Nosé–Hoover–Langevin piston^49^ was used to keep the pressure constant at 1 atm. The pressure was controlled semi-isotropically; the *z* axis of the simulation box, which is normal to the membrane, was allowed to fluctuate independent of the *x* and *y* axes.

**Figure 2:**
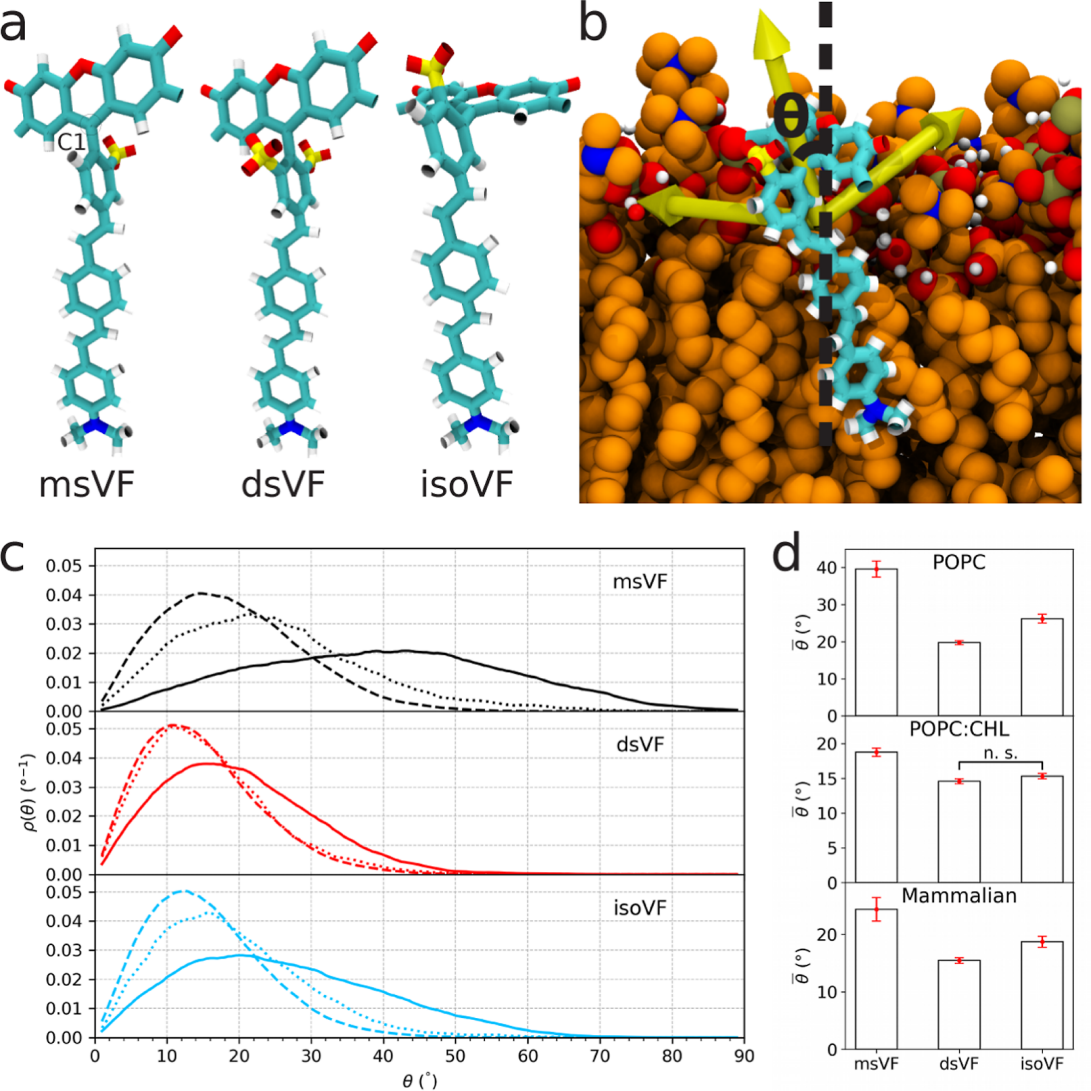
(a) Structures of msVF2.1.Cl, dsVF2.1.Cl, and isoVF2.1.Cl, referred to as msVF, dsVF, and isoVF, respectively. Atom C1 from the fluorescein headgroup is circled in msVF. (b) The tilt angle θ of a VF dye is defined as the angle between its long axis identified via PCA and the membrane normal. (c) Probability density distribution of the tilt angle ρ(θ) of the three VF dyes in POPC (solid), POPC/CHL (2:1) (dashed), and the mammalian membrane (dotted). (d) Average θ  of VF dyes with the standard error of the mean (*e*_θ_) shown as error bars. Statistical significance analyzed by Welch’s *t*-test yields *P* < 0.05 for all pairs of VF dyes in a given lipid environment except for the dsVF–isoVF pair in POPC/CHL, which is labeled by *n.s.* (not significant).

With the membrane placed in the *xy* plane, the orientation of a given VF dye can be characterized by the angle θ between its long axis identified via principal component analysis (PCA) and the *z* axis. Alternatively, θ can be taken as the angle between the *z* axis and a vector pointing from the COM of the molecular wire to that of the sulfofluorescein headgroup, or, the angle between the *z* axis and a vector pointing from the aniline nitrogen to the fluorescein C1 atom (Figure 2). As shown in Figure S1, the three approaches of computing the tilt angle θ yield similar probability density distributions. While the PCA measurement was used in the orientation analysis of plain MD trajectories, for the ease of computation, the COM approach was adopted in the eABF runs. The mean  and standard error of the mean (*e*_θ_) of θ were computed by combining all replicas of plain MD simulations for a given VF dye–lipid system. Specifically, the statistical inefficiency, *g* = 2τ + 1, where τ stands for the integrated autocorrelation time, was first obtained from the time series of θ of each 500 ns simulation trajectory using the pymbar package.^50,51^ The individual statistical inefficiency from each trajectory was averaged to yield *g̅* and then used to determine the standard error of the mean as , where , with *N* representing the number of θ measurements from all simulations of a given VF dye–lipid system and  representing the effective number of uncorrelated measurements. For two-dye systems, measurements from the upper and lower leaflets were both included in the estimation of θ̅ and *e*_θ_. Welch’s *t*-test (two-tailed) was performed using *e*_θ_ and *N*_eff_ via the SciPy library^52^ to compute the *t*-statistic and the corresponding degree of freedom, which were then used to distinguish whether the difference in θ̅ between two VF dyes in the same lipid environment is significant at the 0.05 level (*P* < 0.05). Following our previous work,^37^ the 2D radial pair distribution function *g*(*r*) was measured between the fluorescein C1 atom of VF dyes and the phosphorus (P) atoms of POPC or the oxygen (O) atoms of cholesterol. A resolution of 0.5 Å was adopted, and results were averaged first over a single 500 ns simulation and then over the three replicas. Results of the POPC/CHL bilayer were obtained using the two-dye system with results from the upper and lower leaflets calculated separately and subsequently averaged to produce the final *g*(*r*).

## Results

### Orientation Comparison of msVF, dsVF, and isoVF

As suggested by eq 1, the voltage sensitivity of a given VF dye is closely dependent on its alignment with the membrane normal. In order to probe the influence of the dye structure and lipid composition on such alignment, we examined the orientation of three VF dyes (Figure 2) in three model bilayers: pure POPC, a POPC/CHL (2:1) mixture, and a mammalian membrane. For each VF dye in a given membrane environment, three replicas of 500 ns simulations were performed, resulting in an aggregated 13.5 μs sampling time. The computed average and standard error of the tilt angle θ (see Methods) are listed in Table 1, out of which the pure POPC results are found to be in close agreement with those reported previously.^16,18^ Further analysis of Table 1 as well as the probability density distribution ρ(θ) shown in Figure 2c,d reveals a clear ranking of the three VF dyes: msVF, which has a single sulfonate group at the *meta* position of the aniline donor, is the most heavily tilted in all three model bilayers; dsVF is consistently the least tilted; and isoVF, which is structurally identical to msVF except for the transposed sulfonate and xanthene, ranks between msVF and dsVF, although the difference between isoVF and dsVF is insignificant in the POPC/CHL bilayer. It is worth adding that the same ranking is observed not only in the average of the tilt angle, θ̅, but also in its spread, as shown in Figure 2. In other words, in all lipid environments, msVF and dsVF have the broadest and the narrowest distributions of θ, respectively.

**Table 1:** Average  ± Standard Error (*e*_θ_) of the Tilt Angle θ Measured from Three Replicas of 500 ns Simulations of a VF Dye in a Given Lipid Environment^a^

	θ̅ ± *e*_θ_, τ		
membrane	msVF	dsVF	isoVF
POPC	39.6° ± 2.2°, 11.2 ns	19.8° ± 0.6°, 2.1 ns	26.2° ± 1.2°, 5.8 ns
POPC/CHL	18.8° ± 0.6°, 5.5 ns	14.6° ± 0.3°, 2.8 ns	15.3° ± 0.4°, 3.3 ns
mammalian	24.4° ± 2.0°, 19.2 ns	15.5° ± 0.5°, 2.2 ns	18.7° ± 0.9°, 6.4 ns

a The integrated autocorrelation time τ (ns) averaged over simulations of a given VF dye–lipid system is also provided.

**Figure 3:**
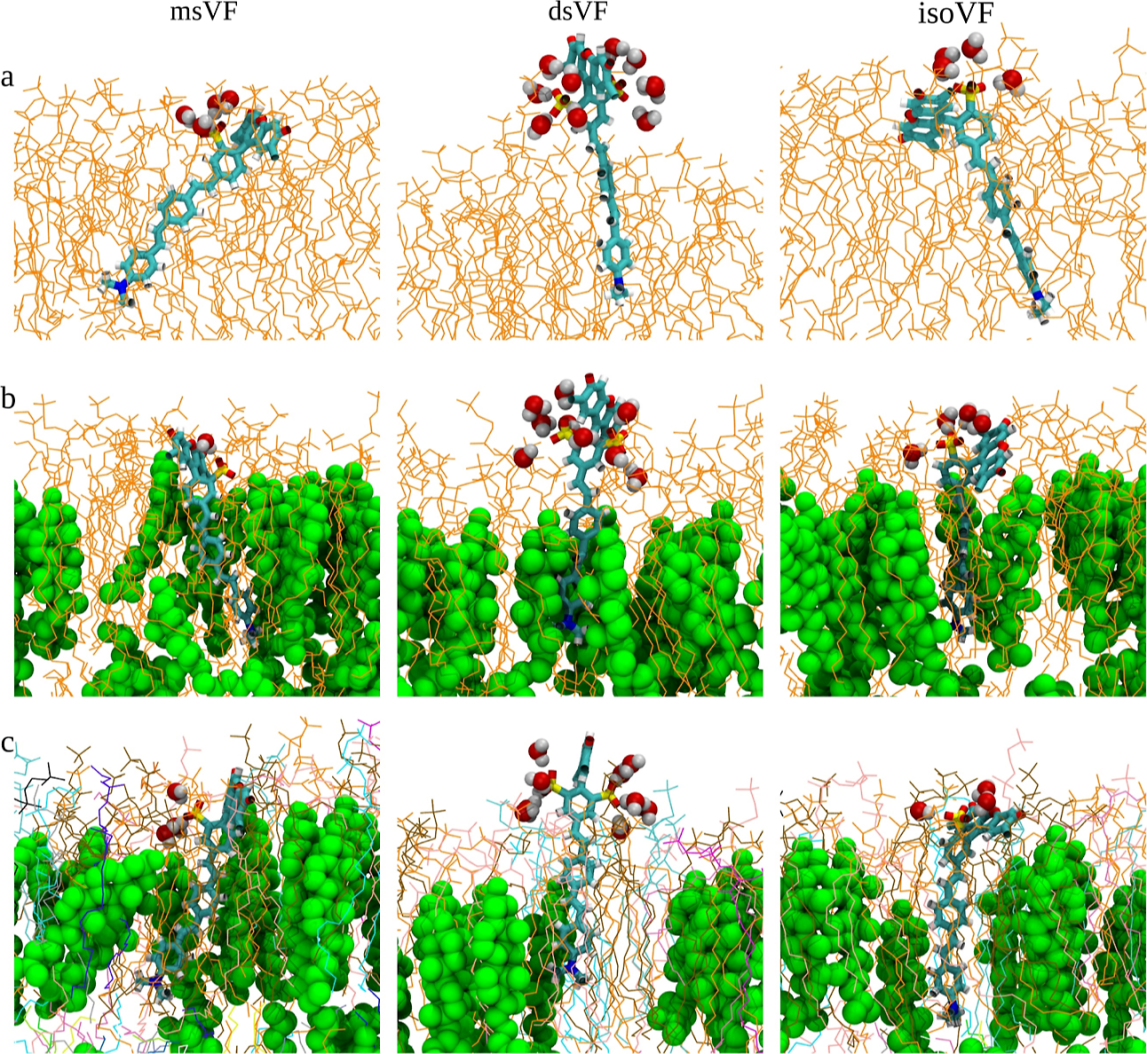
Representative simulation snapshots of VF dyes in pure POPC (a), POPC/CHL (2:1) (b), and the mammalian membrane (c). Cholesterol molecules are highlighted in green vdW representations, while POPC and other lipid species (in the mammalian membrane) are shown in line representations.

Examination of MD trajectories reveals that the VF dyes stayed at the lipid–water interface throughout the simulations, with their charged sulfonates exposed to water and hydrophobic molecular wires buried in the lipid tails of the bilayer (Figure 3). In order to simultaneously meet both requirements, i.e., hydrating the sulfonate(s) and shielding the molecular wire, each VF dye seems to adopt a different strategy based on its individual structure, resulting in the difference in their tilt angle distributions. Specifically, the asymmetrically positioned sulfonate off the main axis in msVF dictates that the molecule needs to tilt substantially in order to avoid partially burying this negatively charged group in the unfavorable environment of the lipid tails. In contrast, for dsVF with two symmetrically positioned sulfonates, tilting is no longer desirable since this cannot solvate both groups at the same time. Instead, the molecule raises itself up in the bilayer—its COM position is 5–6 Å  higher than msVF or isoVF (Table S1), which enables the hydration of both sulfonates at the expense of reduced hydrophobic contact between its molecular wire and the lipid bilayer. As will be discussed later, such reduced hydrophobic contact appears to contribute to the decreased affinity of dsVF toward a membrane. Finally, by transposing the sulfonate and the xanthene groups, isoVF can expose the former to water at a smaller tilt angle than msVF, although the entire molecule remains largely asymmetric, which hinders further reduction in its tilting and ranks isoVF between msVF and dsVF in the distributions of θ.

### Lipid Composition Dependence of VF Dye Orientation

Comparison of simulation results obtained for POPC, POPC/CHL (2:1), and the mammalian membranes clearly reveals the significant impact of lipid composition on VF dye orientation. Specifically, all three VF dyes are found to have reduced θ̅ values in the POPC/CHL mixture relative to the pure POPC bilayer. This reduction is of the largest magnitude in msVF, where θ̅ drops from 39.6 to 18.8°. These changes can be readily associated with the well-established effect of cholesterol in “ordering” a lipid membrane—tilting becomes unfavorable as the order parameter of the lipid tails increases upon the presence of cholesterol, forcing a VF dye to “stand up” and restricting its fluctuation in θ. Out of the three VF dyes, dsVF experiences the smallest change in orientation, moving from pure POPC to a mixed POPC/CHL bilayer. Its raised position in the membrane appears to enable closer packing with the lipids, especially cholesterol, as reflected in the 2D radial distribution function *g*(*r*) shown in Figure S2. Computed between the fluorescein C1 atom from each VF dye and the P atom of POPC or the O atom of cholesterol, the *g*(*r*) profiles from the three VF dyes and their representative conformations in Figure 3 suggest that relative to msVF and isoVF, cholesterol is more closely packed against the hydrophobic molecular wire of dsVF underneath the latter’s bulky and raised sulfofluorescein headgroup.

The mammalian membrane contains a large variety of lipid species, such as phospholipids, cholesterol, sphingolipids, as well as polyunsaturated lipids. It is therefore of interest to examine the orientation of VF dyes within this complex physiologically most relevant environment. As described in the Methods section, all VF dyes were inserted into the outer leaflet of an asymmetric mammalian membrane model, corresponding to their desired location in experimental measurements of the transmembrane potential.^3^ While the different lipid compositions render it impractical to directly compare the “mixing ratio” of the three lipid bilayers studied here, an estimate of the relative cholesterol content can be made; cholesterol is approximately 30 mol % with a number density of ∼0.7 nm^–2^ in both POPC/CHL (2:1) and the outer leaflet of our mammalian membrane model, although the latter system is considerably more heterogeneous in its overall lipid composition. Compared with pure POPC, the similar cholesterol content of POPC/CHL (2:1) and the mammalian membrane suggests that VF dye orientations within the latter two bilayers should bear a closer resemblance to each other. This result is clearly revealed by Figure 2 and Table 1, as well as the overlapping index, which is a number ranging from 0 to 1 that measures the degree of overlap between the ρ(θ) distributions obtained from the three types of membranes, as listed in Table S2. The overlapping index between POPC/CHL and the mammalian membrane exceeds 0.8 for all three VF dyes and consistently ranks above that between pure POPC and the mammalian membrane. However, as noted earlier, while the ranking of θ̅ of the three VF dyes is largely preserved across all three bilayer systems, the difference between dsVF and isoVF in the POPC/CHL bilayer is not significant at the 0.05 level according to Welch’s *t*-test, in contrast to their difference in the mammalian membrane, the latter of which is significant at the same level. This result suggests that attention should be paid when choosing between pure POPC and POPC/CHL (2:1) to probe the tilting tendency of different VF dyes within a mammalian membrane.

### Partition Free Energy of VF Dyes to POPC and POPC/CHL

The amphiphilic scaffold of a VF dye features a long hydrophobic tail that facilitates its insertion into a lipid membrane. While the hydrophobicity of this tail is understandably important to membrane partitioning, the role played by the hydrophilic headgroup of a VF dye is less obvious. The three VF dyes investigated here differ only in their sulfofluorescein headgroup structures. After verifying convergence of eABF calculations (Figures S3 and S4), we summarized Δ*G*_tot_ in Table 2, where it can be seen that the affinity of a VF dye toward a given lipid bilayer strongly depends on the number of sulfonates within its headgroup. Specifically, msVF and isoVF have Δ*G*_tot_ in the range of ∼−9 to −8 kcal/mol and the two VF dyes have approximately the same affinity (up to 0.4 kcal/mol difference) toward a given bilayer, indicating that transposing the chemical groups, i.e., turning an msVF into an isoVF, does not alter the dye’s membrane partition significantly. In clear contrast, the addition of a second sulfonate, i.e., turning an msVF into a dsVF, weakens the membrane binding substantially. As shown in Table 2, dsVF has a Δ*G*_tot_ of only ∼−6 kcal/mol. Compared to msVF and isoVF, the 2–3 kcal/mol loss in Δ*G*_tot_ of dsVF translates to an approximately two-orders-of-magnitude decrease in its insertion probability into a given membrane. Additionally, the minimum of dsVF *W*(*z*) is located further away from the bilayer center, consistent with the profiles of ρ(*z*) obtained from equilibrium simulations (Figure S5). Between the two model bilayers, our data reveal only a slight dependence of Δ*G*_tot_ on lipid compositions for msVF and isoVF, where the two monosulfonate dyes prefer POPC over POPC/CHL by up to 0.8 kcal/mol. Given the minimal lipid composition dependence of Δ*G*_tot_ and the similarity in VF dye orientations between POPC/CHL (2:1) and the mammalian membrane, no additional free energy calculation was pursued for the last system.

**Table 2:** Partition Free Energy Components of VF Dyes

	POPC			POPC/CHL (2:1)		
Δ*G* (kcal/mol)	msVF	dsVF	isoVF	msVF	dsVF	isoVF
Δ*G*_p_^bound^	0.6	0.5	0.6	0.6	0.5	0.6
Δ*G*_c_^bound^	9.1	8.8	8.8	8.2	8.6	8.9
Δ*G*_o_^bound^	1.0	0.9	1.0	0.7	0.8	0.7
Δ*G*_*z*_	–8.8	–6.7	–9.2	–10.0	–7.4	–9.3
Δ*G*_c_^free^	8.2	8.6	8.5	8.2	8.6	8.5
Δ*G*_o_^free^	2.1	2.5	2.3	2.8	2.9	2.8
Δ*G*_tot_	–9.2	–5.9	–8.8	–8.4	–5.9	–8.2

**Figure 4:**
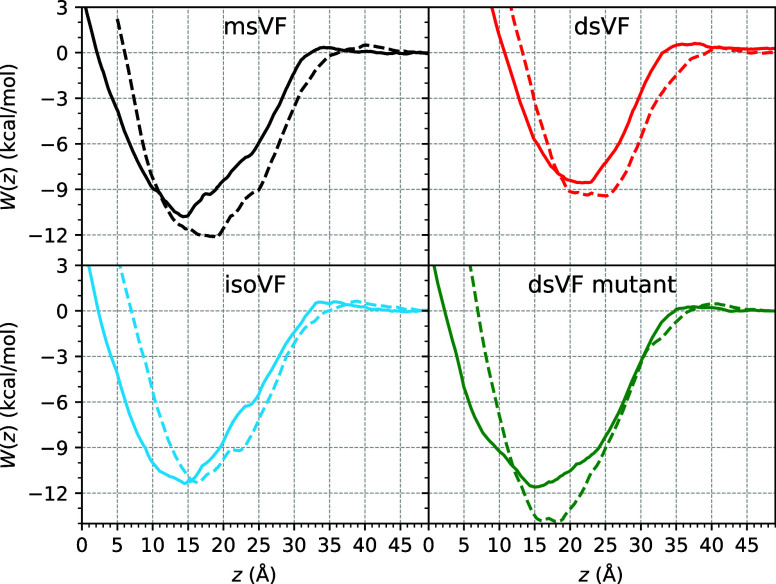
Membrane partition PMF *W*(*z*) of a conformationally and orientationally restrained VF dye to a pure POPC (solid) and a mixed POPC/CHL (2:1) membrane (dashed) for msVF, dsVF, isoVF, and a dsVF mutant with reduced net charge.

In order to identify the physical properties that dominate the distinct membrane partition of the VF dyes, we examined individual contributions to Δ*G*_tot_. By regrouping various contributions, one finds an immobilization free energy cost that has to be paid for the reduced orientational freedom when VF dyes are inserted into the membrane: Δ*G*_o_^free^ – Δ*G*_o_^bound^ ≈ 1–2 kcal/mol. This immobilization cost is 2.1–2.2 kcal/mol for the three VF dyes in POPC/CHL and ranges from 1.1 to 1.6 kcal/mol in pure POPC. The up to ∼1 kcal/mol higher orientational immobilization cost for a given VF dye in the former bilayer contributes to the (small) preference of msVF and isoVF for the latter membrane, where they “tilt more” and enjoy greater orientational freedom; the more symmetric dsVF, in contrast, prefers a less tilted orientation, and its reduced discrimination against POPC/CHL is further balanced by a more favorable Δ*G*_*z*_, resulting in its identical affinity toward the two lipid bilayers. It should be added, however, that the orientational immobilization energy differs by only up to 0.5 kcal/mol between the VF dyes in a given lipid environment and therefore cannot fully account for their differences in Δ*G*_tot_ within the same membrane. The free energy cost associated with the conformational change of VF dyes upon binding, i.e., Δ*G*_c_^free^ – Δ*G*_c_^bound^, is close to zero or negative with a magnitude up to 0.8 kcal/mol. The negative sign indicates that the dye enjoys (slightly) more conformational freedom within a membrane than in bulk water, which likely arises from the favored insertion of the molecular wire into a hydrophobic environment. In POPC, dsVF differs by ∼0.6 kcal/mol in Δ*G*_c_^free^ – Δ*G*_c_^bound^ from msVF, which contributes partly to their differences in Δ*G*_tot_. Finally, for Δ*G*_*z*_, the partition free energy of a geometrically restrained VF dye determined from *W*(*z*), dsVF can be ∼2.5 kcal/mol less favorable than msVF and isoVF, which is also reflected in its shallowest *W*(*z*) energy well within a given membrane (Figure 4). The difference in Δ*G*_*z*_, therefore, emerges as the main contributor to the reduced membrane partition of dsVF relative to the other two VF dyes.

We hypothesize that the lower membrane partition tendency of dsVF than msVF and isoVF is largely due to the extra negative charge brought by its second sulfonate; to strengthen electrostatic interactions between its two sulfonates and surrounding water, dsVF raises itself up away from the membrane center, which compromises the hydrophobic contact between its molecular wire and the lipid bilayer. To test this hypothesis, we designed a “mutant” dsVF with the charges of its two sulfonates each reduced by half, giving the molecule a net charge of −2, instead of the −3 charge of the “native” dsVF. Two additional sets of eABF calculations were then launched to compute the binding PMFs of this mutant dsVF, which was inserted in either a pure POPC or a POPC/CHL (2:1) mixture and transferred reversibly between the bulk solution and the membrane subjected to the same set of restraining potentials as the native dsVF. This mutant dsVF carries the same net charge as msVF and isoVF, and, therefore, is less hydrophilic than the native dsVF. As revealed by the corresponding *W*(*z*) in Figure 4, the mutant dsVF now has an energy minimum comparable to that of msVF and isoVF in pure POPC, while in the POPC/CHL mixture, the mutant dsVF *W*(*z*) has an energy minimum about ∼2 kcal/mol more favorable than that of msVF and isoVF. These results verify our hypothesis and confirm that the weaker membrane affinity of dsVF than msVF and isoVF can be largely attributed to the strong electrostatic interactions with water brought by its double sulfonates.

## Discussion

In this work, we conducted extensive MD simulations to determine the orientation of three VF dyes within different lipid environments and then employed eABF calculations to quantify their partition in model bilayers. Our results show that the orientation of a VF dye depends on its molecular structure as well as the lipid composition of the membrane it is embedded in. Among the three VF dyes investigated here, the consistent ranking of msVF > isoVF and msVF > dsVF is recorded in their tilt angle θ within all three bilayers. IsoVF is found to rank between msVF and dsVF in pure POPC and the mammalian membrane, while in POPC/CHL, the difference between isoVF and dsVF is insignificant. Such ranking is similarly reflected in the variance of θ, i.e., the VF dyes that tilt less also tend to fluctuate less in their orientations (Figure 2). These results, along with the structural comparison of the three VF dyes, suggest that the symmetry in their molecular scaffolds strongly affects the alignment of these fluorescent probes with the membrane normal. From a rational design perspective, this alignment can be improved by placing components within the bulky headgroup of a VF dye, especially those carrying net charges, as symmetrically as possible—for dsVF, this means placing both of its sulfonates at the *meta* positions of the aniline donor and for isoVF, this corresponds to placing the single sulfonate at the *para* position.

Between the two commonly used model bilayers, namely, pure POPC and a POPC/CHL (2:1) mixture, the latter consistently bears closer resemblance to a mammalian membrane in terms of the tilt angle measured for a given VF dye. The similarity in results obtained from the latter two membranes suggests that a POPC/CHL mixture may substitute the more complex mammalian membrane in the study of VF dye orientation, although if one is interested in ranking different dyes, the trend in the comparison of the mean value as well as variance of θ within the mammalian membrane may be equally well or even better preserved by the pure POPC bilayer. It is worth adding, however, that the voltage sensitivity of a VF dye depends on a number of factors, such as the energy gap between the molecular orbitals of the electron transfer donor and acceptor.^18^ Therefore, while the tilt angle in a lipid membrane is known to play an important part,^16^ it is certainly not the only consideration in the design of these fluorescent probes.

One additional factor that may contribute to the differential performance of VF dyes is their partition into a lipid membrane. By design, the hydrophilic/charged fluorescein head paired with a hydrophobic molecular wire provides a VF dye with the amphiphilicity needed for incorporation into the membrane. Since only those dyes successfully inserted can respond to transmembrane potential variation, a greater membrane partition should yield a stronger signal from the lipid bilayer at a given bulk concentration of VF dyes. From our free energy calculations of msVF, dsVF, and isoVF as well as a “dsVF mutant”, the net charge on the sulfonate group(s) emerges as a major contributor to the difference in their membrane partition. The −2*e* charge borne by its two sulfonates and the resulting solvation by surrounding water molecules effectively “unplug” the molecular wire of dsVF from the bilayer core, thereby, weakening its membrane affinity. Artificially cutting the net charge of its two sulfonates by half significantly strengthened the membrane binding of the dsVF mutant. Thus, reducing the net charge of their hydrophilic headgroups appears to be an effective strategy to improve the membrane partition of these amphiphilic probes.

During the computation of Δ*G*_tot_, a simulation strategy involving multiple restraining potentials^23−25,31^ was employed, where the series of restraints were introduced to speed up the convergence. Compared with the original scheme designed for protein–ligand or protein–protein binding, the lateral homogeneity of the membrane system significantly reduces the number of required collective variables and a simpler form of the orientational restraint *u*_o_ can be employed. An additional positional restraint, *u*_p_, however, had to be introduced to confine the VF dyes in their most probable location within the membrane. While it was not present in the original protocol of Woo and Roux^23^ or subsequent calculations using the PMF approach by Gumbart et al.,^24^*u*_p_ is analogous to the *u*_r_ term in the alchemical approach by the latter authors, where the extra restraint was introduced to confine a ligand’s COM position within the binding site of a receptor. This flat-bottom *u*_p_ restraint limits the space explored by the dyes, thus, accelerating the convergence of PMF calculations along other degrees of freedom, such as RMSD and θ. The adoption of *u*_p_ introduces an exponential term  into eqs 5 and 6. Due to this exponential term, the integral in both equations is dominated by the region with *u*_p_ = 0, making the exact range of integration defined by *z*_B-L_ and *z*_B-U_ unimportant as long as this range contains the entire *u*_p_ ≈ 0 region. Nonetheless, the thickness of the bound domain, defined by *z*_B-U_ – *z*_B-L_, enters eq 6 as a denominator. As noted earlier, with *z*_B-U_ – *z*_B-L_ equal to the membrane thickness, Δ*G*_tot_ represents the partition free energy of a VF dye between the lipid and water phases. It should be added that by restricting *z*_p-L_ and *z*_p-U_ to the peak region of ρ(*z*) obtained from a single monolayer, we assume that VF dye insertion occurs primarily in one leaflet of the lipid membrane (the outer leaflet of the plasma membrane). In other words, the barrier against VF dye “flip flop” is assumed to be sufficiently high to render its partition into the second monolayer insignificant on the experimental time scale. This assumption is in line with the design principle and performance of VF dyes^3^ and can be largely attributed to the −2 or −3 net charges they carry. That being said, if the partition of a VF dye is to be considered for both monolayers, one may simply set *z*_B-U_ – *z*_B-L_ to half of the membrane thickness (this will produce a decrease of ∼0.4 kcal/mol in Δ*G*_*z*_), while still computing *W*(*z*) for only a single monolayer to take advantage of a bilayer’s symmetry.

An alternative, powerful route for computing the binding free energy of biomolecules is the double-decoupling scheme^53^ employing the free energy perturbation (FEP) method, in which the molecules of interest in the bound and the free states are each decoupled from the surrounding medium and the obtained free energy difference yields Δ*G*_tot_. Unfortunately, this scheme poses considerable challenges for VF dyes, given their sizes and polarities. Furthermore, the net charges carried by these dyes present an electrostatic finite-size effect, the elimination of which would require additional computation.^54^ While the PMF approach is better suited than the FEP approach for VF dyes, it is worth adding that the PMFs shown in Figure 4 do not depict the energy landscape of a free VF dye crossing a lipid bilayer. These PMFs were determined in the presence of restraints on both RMSD and θ, the contributions of which had to be accounted for to yield the final Δ*G*_tot_. As a result, while it may be tempting to estimate the transmembrane free energy barrier against a VF dye at *z* = 0 from Figure 4, this value is unlikely to be accurate given that it corresponds to a conformationally and orientationally restrained VF dye. Accurate measurement of this barrier, albeit beyond the scope of this study, should provide additional information on the intracellular distribution of these membrane-bound probes—those with a large transmembrane barrier are more likely to reside in the outer leaflet of the plasma membrane, whereas those with a small barrier may readily permeate the plasma membrane and subsequently embed themselves into various organelle membranes. For the VF dyes studied here, which were designed to sense the voltage change across the plasma membrane, a low transmembrane energy barrier is to be avoided since dyes that readily “flip flop” into the inner leaflet of the plasma membrane will have a reduced signal-to-noise ratio. Nonetheless, for other molecular probes targeting intracellular organelles, such as the recently developed SPIRIT RhoVR,^17^ a low transmembrane energy barrier can become desirable instead.

## Conclusions

To elucidate how physicochemical properties of VF dyes govern their membrane orientation and partition, we conducted microsecond MD simulations on three VF dyes in pure POPC, mixed POPC/CHL, as well as a mammalian membrane model. Our data reveal that the orientation of a VF dye strongly depends on its own structural symmetry as well as the lipid composition of the membrane it is embedded in. Switching the single sulfonate from the *meta* position of the aniline donor in msVF to the *para* position in isoVF enables the latter molecule to better align itself with the membrane normal, while the even more symmetric arrangement of two sulfonates in dsVF further improves such alignment. The presence of cholesterol, which is about 30 mol % in both our POPC/CHL (2:1) and the mammalian membrane models, significantly reduces the tilt angles of all VF dyes. Affinity of the dyes toward POPC and POPC/CHL shows slight (up to 0.8 kcal/mol) dependence on lipid composition in msVF and isoVF, both of which prefer the former lipid environment where they enjoy greater orientational freedom. Within the same lipid environment, the single property that contributes the most to a 2–3 kcal/mol weaker Δ*G*_tot_ of dsVF than the other two VF dyes appears to be its headgroup’s net charge. Reducing this net charge while maintaining the symmetry of the molecular scaffold may therefore be considered in the future design of VF dyes with both good voltage sensitivity and strong membrane partition. In calculating Δ*G*_tot_ for VF dyes, we find that restraining simulation protocols designed for protein–ligand or protein–protein binding can be well adapted and employed, which should be equally applicable in quantifying the partition tendency of other membrane-bound, small-molecule-based probes.

## Supporting Information Available

Details on Δ*G*_tot_ calculation, eABF simulation, and analysis protocols (PDF)
