# Supplementary material for: Orientation and Membrane Partition Free Energy of PeT-Based Voltage-Sensitive Dyes from Molecular Simulations
Source: J Phys Chem B. 2024 Mar 9;128(11):2734–44. doi: 10.1021/acs.jpcb.3c08090 (PMC10961725; doi:10.1021/acs.jpcb.3c08090)
Supplement: Supplementary file 1 — jp3c08090_si_001.pdf [file jp3c08090_si_001.pdf]

Orientation and Membrane Partition Free Energy of PeT-Based Voltage-Sensitive Dyes from Molecular Simulations

Chun Kei Lam, Lap Yan Fung and Yi Wang

Department of Physics, The Chinese University of Hong Kong, Shatin, Hong Kong SAR, China

Supporting Information

Membrane partition free energy of VF dye

As noted in the Methods section, we consider a system with a lipid bilayer of lateral surface area A placed in a water box (Fig. 1) and assume that a total of N identical VF dyes are present, out of which N_b partition into the membrane (bound) and N_f are in the solution (free). In the limit of low concentration, the chemical potential of bound and free VF dyes are:[27,53]

$$\begin{aligned} \mu_b &= \mu_b^\circ + k_B T \ln \frac{C_b}{C^\circ} \\ \mu_f &= \mu_f^\circ + k_B T \ln \frac{C_f}{C^\circ}, \end{aligned} \tag{S1}$$

where μ_i° and C_i are the standard chemical potential and the concentration of the VF dyes in the bound state ($i = b$) and the free state ($i = f$), respectively, while C° is the standard concentration with the same unit as C_b and C_f. At equilibrium, the chemical potential in the two states must be equal, *i.e.*, $\mu_b = \mu_f$. The membrane partition free energy of the VF dye is therefore given by:

$$
\begin{aligned}
\Delta G_{\text{tot}} &\equiv \mu_b^\circ - \mu_f^\circ \\
&= -k_B T \ln \Big(\frac{C_b}{C_f}\Big)_{eq} \\
&= -k_B T \ln \Big(\frac{\frac{N_b}{V_b}}{\frac{N_f}{V_f}}\Big)_{eq} \\
&= -k_B T \ln \frac{\frac{1}{V_b}\int_{\text{bound}} d\mathbf{1} \int d\mathbf{X} e^{-\beta U}}{\frac{1}{V_f}\int_{\text{free}} d\mathbf{1} \int d\mathbf{X} e^{-\beta U}} \\
&= -k_B T \ln \frac{\frac{1}{V_b}\int_{\text{bound}} d\mathbf{1} \int d\mathbf{X} e^{-\beta U}}{\int_{\text{free}} d\mathbf{1} \int d\mathbf{X} \delta(\mathbf{r}_1 - \mathbf{r}_1^*) e^{-\beta U}},
\end{aligned}
\tag{S2}
$$

where the subscript eq indicates equilibrium state, while V_b and V_f denote the volume of the bound domain and the free solution, respectively. As in ref,[23] the homogeneity of bulk solution is invoked to recast the denominator in the last line. Following a previously developed simulation strategy[23,24,31,33] that employs a series of restrained intermediate states, we further compute ΔG_{tot} according to:

$$
\begin{aligned}
e^{-\beta \Delta G_{\text{tot}}} = &\frac{\int_{\text{bound}} d\mathbf{1} \int d\mathbf{X} e^{-\beta U}}{\int_{\text{bound}} d\mathbf{1} \int d\mathbf{X} e^{-\beta [U + u_{\text{p}}]}} && \text{(S3a)} \\
&\times \frac{\int_{\text{bound}} d\mathbf{1} \int d\mathbf{X} e^{-\beta [U + u_{\text{p}}]}}{\int_{\text{bound}} d\mathbf{1} \int d\mathbf{X} e^{-\beta [U + u_{\text{p}} + u_{\text{c}}]}} && \text{(S3b)} \\
&\times \frac{\int_{\text{bound}} d\mathbf{1} \int d\mathbf{X} e^{-\beta [U + u_{\text{p}} + u_{\text{c}}]}}{\int_{\text{bound}} d\mathbf{1} \int d\mathbf{X} e^{-\beta [U + u_{\text{p}} + u_{\text{c}} + u_{\text{o}}]}} && \text{(S3c)} \\
&\times \frac{\frac{1}{V_b}\int_{\text{bound}} d\mathbf{1} \int d\mathbf{X} e^{-\beta [U + u_{\text{p}} + u_{\text{c}} + u_{\text{o}}]}}{\int_{\text{free}} d\mathbf{1} \delta(\mathbf{r}_1 - \mathbf{r}_1^*) \int d\mathbf{X} e^{-\beta [U + u_{\text{c}} + u_{\text{o}}]}} && \text{(S3d)} \\
&\times \frac{\int_{\text{free}} d\mathbf{1} \delta(\mathbf{r}_1 - \mathbf{r}_1^*) \int d\mathbf{X} e^{-\beta [U + u_{\text{c}} + u_{\text{o}}]}}{\int_{\text{free}} d\mathbf{1} \delta(\mathbf{r}_1 - \mathbf{r}_1^*) \int d\mathbf{X} e^{-\beta [U + u_{\text{c}}]}} && \text{(S3e)} \\
&\times \frac{\int_{\text{free}} d\mathbf{1} \delta(\mathbf{r}_1 - \mathbf{r}_1^*) \int d\mathbf{X} e^{-\beta [U + u_{\text{c}}]}}{\int_{\text{free}} d\mathbf{1} \delta(\mathbf{r}_1 - \mathbf{r}_1^*) \int d\mathbf{X} e^{-\beta U}}. && \text{(S3f)}
\end{aligned}
$$

The above set of equations insert a series of intermediate states into Eq. S2 to compute ΔG_{tot}: a positional (u_{p}), conformational (u_{c}) and orientation (u_{o}) restraining potential is imposed sequentially onto a VF dye in the bound state, which is then transferred reversibly

from the lipid membrane to the free solution, where the imposed restraints are removed one by one. Eq. S3a to Eq. S3f correspond to the six components of $\Delta G_{\rm tot}$ given by Eq. 3 in the Methods section. With $\Delta G_{\rm p}^{\rm bound}$ and ΔG_z described therein, below we provide details for the remaining four components as well as derivation of the equation used to compute ΔG_z (Eq. 6).

$\Delta G_{\rm c}^{\rm bound}$ from conformational restraint in the bound state The conformational restraint $u_{\rm c}$ imposed on a bound VF dye in the presence of $u_{\rm p}$ results in a free energy cost $\Delta G_{\rm c}^{\rm bound}$ given by

$$\begin{aligned} e^{-\beta \Delta G_{\rm c}^{\rm bound}} &\equiv \frac{\int_{\rm bound} d\mathbf{1} \int d\mathbf{X} e^{-\beta[U+u_{\rm p}+u_{\rm c}]}}{\int_{\rm bound} d\mathbf{1} \int d\mathbf{X} e^{-\beta[U+u_{\rm p}]}} \\ &= \frac{\int_{\rm bound} d\xi e^{-\beta W(\xi)} e^{-\beta u_{\rm c}(\xi)}}{\int_{\rm bound} d\xi e^{-\beta W(\xi)}} \\ &= \langle e^{-\beta u_{\rm c}} \rangle_{({\rm bound}, u+u_{\rm p})}. \end{aligned} \tag{S4}$$

The conformational restraint used in this work is identical to the term $u_{\rm c}$ employed in earlier protein-ligand[23] or protein-protein[31] binding free energy calculations. Here, the geometrically optimized structure of the VF dyes, prepared by our lab in a previous study,[16] was used as the reference structure for calculating the RMSD ξ of atomic positions of the VF dye. The conformational restraint $u_{\rm c}$ has a harmonic form: $u_{\rm c} = \frac{1}{2}k_{\rm c}\xi^2$, where $k_{\rm c}$ is the force constant of the restraint. Evaluation of the above equation is achieved through determining $W(\xi)$, the PMF along ξ in the presence of $u_{\rm p}$, via the eABF method (Fig. S6). Parameters used in this and other eABF calculations are provided in the last section of this Supporting Information.

$\Delta G_{\rm o}^{\rm bound}$ from orientational restraint in the bound state Following the computation of $\Delta G_{\rm c}^{\rm bound}$, subsequent eABF runs of a positionally and conformationally restrained VF dye in the membrane yield the PMF along θ, $W(\theta)$, the minimum of which is used as reference to set θ_0 in the orientational restraint $u_{\rm o} = \frac{1}{2}k_{\rm o}(\theta - \theta_0)^2$, where $k_{\rm o}$ is the spring

constant of the harmonic restraint. Compared with the orientational restraint in protein-ligand association[23] which involved three Euler angles, the u_o term here is much simpler due to the lateral homogeneity of the membrane. The free energy cost $\Delta G_\mathrm{o}^\mathrm{bound}$ of introducing this orientational restraint to a positionally and conformationally restrained VF dye in the bound state is given by:

$$\begin{aligned} e^{-\beta \Delta G_\mathrm{o}^\mathrm{bound}} &\equiv \frac{\int_\mathrm{bound} d\mathbf{1} \int d\mathbf{X} e^{-\beta[U+u_\mathrm{p}+u_\mathrm{c}+u_\mathrm{o}]}}{\int_\mathrm{bound} d\mathbf{1} \int d\mathbf{X} e^{-\beta[U+u_\mathrm{p}+u_\mathrm{c}]}} \\ &= \frac{\int_\mathrm{bound} d\theta e^{-\beta W(\theta)} e^{-\beta u_\mathrm{o}(\theta)}}{\int_\mathrm{bound} d\theta e^{-\beta W(\theta)}} \\ &= \langle e^{-\beta u_\mathrm{o}} \rangle_{(\mathrm{bound}, u+u_\mathrm{p}+u_\mathrm{c})}. \end{aligned} \tag{S5}$$

Evaluation of the above equation is similar to that of Eq. S4 via the eABF method.

ΔG_z as the membrane partition free energy of a geometrically restrained VF dye

To obtain Eq. 6 in the Methods section, we first define a function as in ref:[23]

$$f(\mathbf{r}_1') \equiv \int d\mathbf{1} \delta(\mathbf{r}_1 - \mathbf{r}_1') \int d\mathbf{X} e^{-\beta[U+u_\mathrm{c}+u_\mathrm{o}]}, \tag{S6}$$

where in cylindrical coordinates $\mathbf{r}_1 = (\rho, \phi, z)$. Far away from the membrane, the function $f(\mathbf{r}_1^*) \equiv f(\rho^*, \phi^*, z^*)$ becomes independent of ρ^* and ϕ^*, *i.e.*, $f(\mathbf{r}_1^*) = f(0, 0, z^*)$. ΔG_z can then be obtained as

$$
\begin{aligned}
e^{-\beta\Delta G_z} &\equiv \frac{\frac{1}{V_b}\int_{\text{bound}} d\mathbf{1} \int d\mathbf{X} e^{-\beta[U+u_{\text{p}}+u_{\text{c}}+u_{\text{o}}]}}{\int_{\text{free}} d\mathbf{1}\delta(\mathbf{r}_1 - \mathbf{r}_1^*) \int d\mathbf{X} e^{-\beta[U+u_{\text{c}}+u_{\text{o}}]}} \\
&= \frac{\frac{1}{V_b}\int_{\text{bound}} d\mathbf{r}_1 f(\mathbf{r}_1) e^{-\beta u_{\text{p}}}}{f(\mathbf{r}_1^*)} \\
&= \frac{\frac{1}{V_b}\int_{\text{bound}} d\mathbf{r}_1 f(\mathbf{r}_1) e^{-\beta u_{\text{p}}}}{f(0,0,z^*)} \\
&= \frac{\frac{1}{V_b}\int_{\text{bound}} d\mathbf{r}_1 f(\mathbf{r}_1) e^{-\beta u_{\text{p}}}}{f(0,0,z^*) \times \frac{\int d\mathbf{r}_1 \delta(z-z^*)}{\int d\mathbf{r}_1 \delta(z-z^*)}} \\
&= \frac{A}{V_b} \frac{\int_{\text{bound}} d\mathbf{r}_1 f(\mathbf{r}_1) e^{-\beta u_{\text{p}}}}{f(0,0,z^*) \times \int d\mathbf{r}_1 \delta(z-z^*)} \\
&= \frac{A}{V_b} \frac{\int_{\text{bound}} d\mathbf{r}_1 f(\mathbf{r}_1) e^{-\beta u_{\text{p}}}}{\int d\mathbf{r}_1 f(\mathbf{r}_1)\delta(z-z^*)} \\
&= \frac{A}{V_b} \int_{\text{bound}} dz' \frac{\int d\mathbf{r}_1 f(\mathbf{r}_1)\delta(z-z') e^{-\beta u_{\text{p}}}}{\int d\mathbf{r}_1 f(\mathbf{r}_1)\delta(z-z^*)} \\
&= \frac{A}{V_b} \int_{\text{bound}} dz' e^{-\beta u_{\text{p}}} e^{-\beta[W(z')-W(z^*)]} \\
&= \frac{1}{z_{\text{B-U}} - z_{\text{B-L}}} \int_{z_{\text{B-L}}}^{z_{\text{B-U}}} dz'\, e^{-\beta u_{\text{p}}} e^{-\beta[W(z')-W(z^*)]}
\end{aligned} \qquad \text{(S7)}
$$

where $A = \int d\mathbf{r}_1 \delta(z - z^*)$ is the lateral area of the membrane, and $W(z)$ is the potential of mean force of the VF dye as a function of z in the presence of the conformational restraint u_c and orientational restraint u_o:

$$
e^{-\beta[W(z')-W(z^*)]} = \frac{\int d\mathbf{1}\delta(z-z') \int d\mathbf{X} e^{-\beta[U+u_{\text{c}}+u_{\text{o}}]}}{\int d\mathbf{1}\delta(z-z^*) \int d\mathbf{X} e^{-\beta[U+u_{\text{c}}+u_{\text{o}}]}} \qquad \text{(S8)}
$$

$\Delta G_{\text{o}}^{\text{free}}$ from orientational restraint in the free state Computation of $\Delta G_{\text{o}}^{\text{free}}$ is known to be straightforward thanks to the homogeneity and isotropy of bulk solution.[23] This contribution from the orientational restraint u_{o} applied on a conformationally restrained VF dye in the free state is given by:

$$
\begin{aligned}
e^{-\beta \Delta G_{\mathrm{o}}^{\mathrm{free}}} &\equiv \frac{\int_{\mathrm{free}} d\mathbf{1}\delta(\mathbf{r}_1 - \mathbf{r}_1^*) \int d\mathbf{X} e^{-\beta[U+u_c+u_o]}}{\int_{\mathrm{free}} d\mathbf{1}\delta(\mathbf{r}_1 - \mathbf{r}_1^*) \int d\mathbf{X} e^{-\beta[U+u_c]}} \\
&= \frac{1}{2}\int_0^{\pi} d\theta \sin(\theta) e^{-\beta u_{\mathrm{o}}}
\end{aligned}
\tag{S9}
$$

With the parameters of u_{o} given in Table S3, Eq. S9 is readily evaluated via numerical integration.

$\Delta G_{\mathbf{c}}^{\mathbf{free}}$ from conformational restraint in the free state $\Delta G_{\mathrm{c}}^{\mathrm{free}}$, the free energy cost of introducing the conformational restraint u_c to an unrestrained VF dye in the free state is given by:[23]

$$
\begin{aligned}
e^{-\beta \Delta G_{c}^{\mathrm{free}}} &\equiv \frac{\int_{\mathrm{free}} d\mathbf{1}\delta(\mathbf{r_1} - \mathbf{r_1^*}) \int d\mathbf{X} e^{-\beta[U+u_c]}}{\int_{\mathrm{free}} d\mathbf{1}\delta(\mathbf{r_1} - \mathbf{r_1^*}) \int d\mathbf{X} e^{-\beta U}} \\
&= \frac{\int_{\mathrm{free}} d\xi e^{-\beta W(\xi)} e^{-\beta u_c(\xi)}}{\int_{\mathrm{free}} d\xi e^{-\beta W(\xi)}} \\
&= \langle e^{-\beta u_c} \rangle_{(\mathrm{free,U})}
\end{aligned}
\tag{S10}
$$

Evaluation of the above equation employs the same eABF approach as that of $\Delta G_{\mathrm{c}}^{\mathrm{bound}}$. Note that this term depends on the dye molecule only. Therefore, only a single PMF calculation is performed for each VF dye (Table S4).

eABF simulation and analysis protocols

All PMFs along selected collective variables, namely, $W(\xi)$, $W(\theta)$, and $W(z)$ are determined by the eABF[34,35] method with the corrected z-averaged restraint (CZAR) scheme in Colvar.[36] The order of the PMF computation largely follows that of the corresponding terms in Eq. S3: $W(\xi)$ (Fig. S6) in the bound state is first computed with two eABF windows jointly spanning $0.5\,\text{Å} \leq \xi \leq 2.5\,\text{Å}$ and initial structures obtained from plain MD simulations of the corresponding VF dye in a POPC or POPC:CHL bilayer. The $W(\xi)$ calculation

yields $\Delta G_{\mathrm{c}}^{\mathrm{bound}}$, which is followed by $\Delta G_{\mathrm{o}}^{\mathrm{bound}}$ calculation with $W(\theta)$ (Fig S7) computed via eABF using either a single window spanning $0^\circ < \theta \leq 35^\circ$ (in POPC:CHL) or up to three windows jointly spanning $0^\circ < \theta \leq$ 50-70° (in POPC). The range of θ varies in the POPC system due to different tilting tendencies of the three VF dyes in this bilayer. Note that the exponential term involving u_{o} in Eq. S5 dictates that only the part of $W(\theta)$ (Fig.S7) near the center of u_{o} (Table S3) contributes significantly to $\Delta G_{\mathrm{o}}^{\mathrm{bound}}$. For both POPC and POPC:CHL bilayers, initial structures of the $W(\theta)$ calculation were obtained from the aforementioned $W(\xi)$ eABF runs. Next, to prepare for the eABF calculation of $W(z)$, a 50-ns steered MD (sMD) simulation was performed to pull (force constant: 100 kcal/(mol/Å^2)) the conformationally and orientationally restrained VF dye from $z = 5$ Å to $z = 55$ Å. Subsequent eABF calculations of $W(z)$ were then performed in up to 27 windows spanning the range 0-5 Å $\leq z \leq$ 49-57 Å with initial structures obtained from the above sMD simulations. The range of z depends on the preferred depth of different VF dyes within a membrane. Again, due to the exponential term involving u_{p}, only the part of $W(z)$ near the center of u_{p} (Table S3) makes significant contribution to ΔG_z. Finally, $\Delta G_{\mathrm{c}}^{\mathrm{free}}$ is obtained by computing $W(\xi)$ in the free solution using initial structures obtained from the $W(z)$ eABF runs. Similar as $W(\xi)$ calculation in the bound state, two windows jointly spanning 0.5 Å $\leq \xi \leq$ 2.5 Å were employed. In all eABF calculations, the parameter `extendedTimeConstant` was set to 200 fs. Instantaneous forces were collected in bins of width 0.05 Å, 1°, and 0.1 Å for $W(\xi)$, $W(\theta)$, and $W(z)$, respectively, with the parameter `extendedFluctuation` equal to the bin width. Force constants of positional (k_p), conformational (k_c), and orientational (k_o) restraints were set to 100 kcal/(mol Å^2), 10 kcal/(mol Å^2), and 0.1 kcal/(mol degree^2), respectively, while the center of each restraint is given in Table S3. The total simulation time for each of the above PMF calculations is given in Table S4. Convergence of the eABF calculations was analyzed by computing ΔG_{tot} using an increasing percentage of the total eABF simulations, *i.e.*, at a given sampling percentage s, where $s = 10\%, 20\%, .., 100\%$, all components of ΔG_{tot} were calculated using only the first s percentage of the corresponding eABF runs,

with the exception of $\Delta G_{\mathrm{p}}^{\mathrm{bound}}$ and $\Delta G_{\mathrm{o}}^{\mathrm{free}}$, which, as described earlier, were obtained from equilibrium MD simulations and numerical integration, respectively.

Table S1: Mean value of the membrane thickness, measured as the average distance between phosphorus atoms in the two leaflets, z coordinate of CoM position of VF dye relative to membrane center $\overline{z}_{\mathbf{MemCen}}$, and z coordinate of CoM position of VF dye relative to the average position of upper-layer phosphorus atoms $\overline{z}_{\mathbf{upperP}}$.

system		thickness (Å)	$\overline{z}_{\mathrm{MemCen}}$ (Å)	$\overline{z}_{\mathrm{upperP}}$ (Å)
POPC	msVF	38.6	15.3	-4.2
	dsVF	38.7	21.1	1.5
	isoVF	38.5	15.7	-3.8
POPC/Chol	msVF	44.7	17.0	-5.3
	dsVF	44.8	22.2	-0.1
	isoVF	44.7	16.9	-5.5

Table S2: Overlapping index between $\rho(\theta)$ of a given VF dye measured from two different membranes (POPC-MAMM or POPC:CHL-MAMM). The overlapping index between two distributions $\rho_A(\theta)$ and $\rho_B(\theta)$ is computed as $\int \min|\rho_A(\theta), \rho_B(\theta)|d\theta$, where larger values indicate greater overlap between the two given probability density distributions.

System	POPC-MAMM	POPC:CHL-MAMM
msVF	0.60	0.81
dsVF	0.80	0.96
isoVF	0.75	0.86

Table S3: Parameter values of $z_{\text{p-L}}$, $z_{\text{p-U}}$ and θ_0 used in membrane partition free energy calculations.

system		$z_{\text{p-L}}$ (Å)	$z_{\text{p-U}}$ (Å)	θ_0 (°)
POPC	msVF	14.0	16.0	35.3
	dsVF	20.0	22.0	19.4
	isoVF	15.0	17.0	24.9
POPC/Chol	msVF	15.0	17.0	11.0
	dsVF	21.0	23.0	9.0
	isoVF	15.0	17.0	12.0

Table S4: Simulation time spent on determining various contributions to ΔG_{tot}.

Simulation time (ns)						
	POPC			POPC:CHL		
contribution	msVF	dsVF	isoVF	msVF	dsVF	isoVF
$\Delta G_{\mathrm{c}}^{\mathrm{bound}}$	120	120	120	120	120	120
$\Delta G_{\mathrm{o}}^{\mathrm{bound}}$	200	100	150	150	150	150
ΔG_z	560	550	620	630	750	760
$\Delta G_{\mathrm{c}}^{\mathrm{free}}$	120	120	120	-	-	-
$\Delta G_{\mathrm{o}}^{\mathrm{free}}$	-	-	-	-	-	-
ΔG_{tot}	1000	890	1010	900	1020	1030

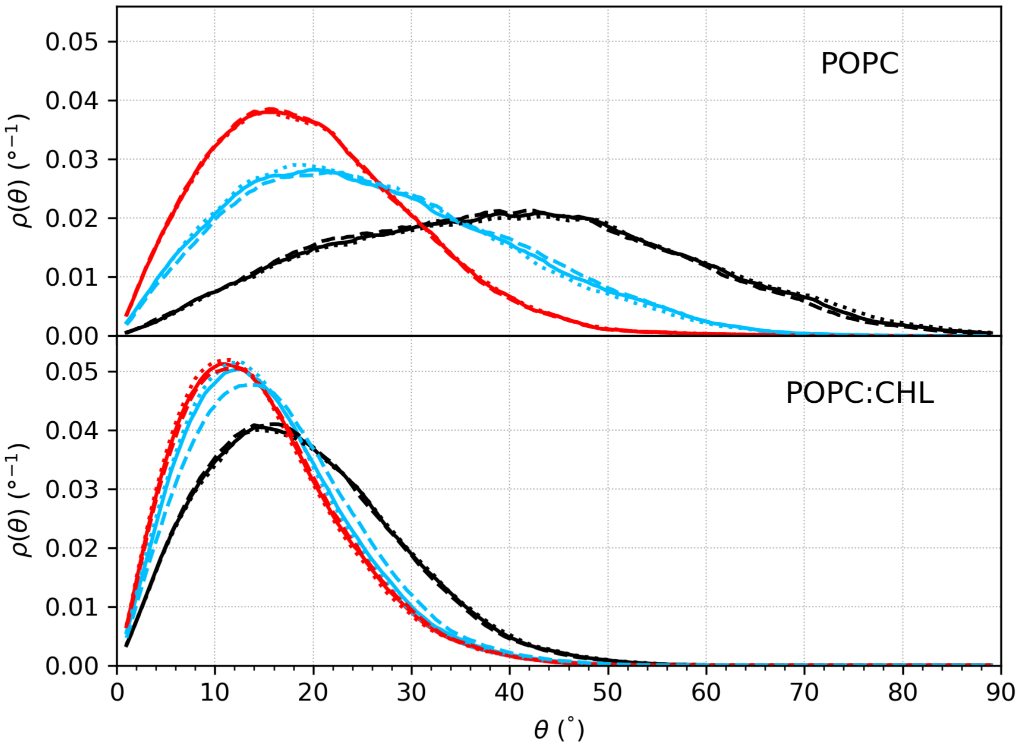

Figure S1: Probability density distribution of the tilt angle $\rho(\theta)$ of msVF (black), dsVF (red) and isoVF (cyan) computed from principle component analysis (solid), the vector connecting the aniline nitrogen and the fluorescein C1 atom (dotted), or the vector connecting the center-of-mass locations of the sulfofluorescein and the rest of the dye molecule (dashed).

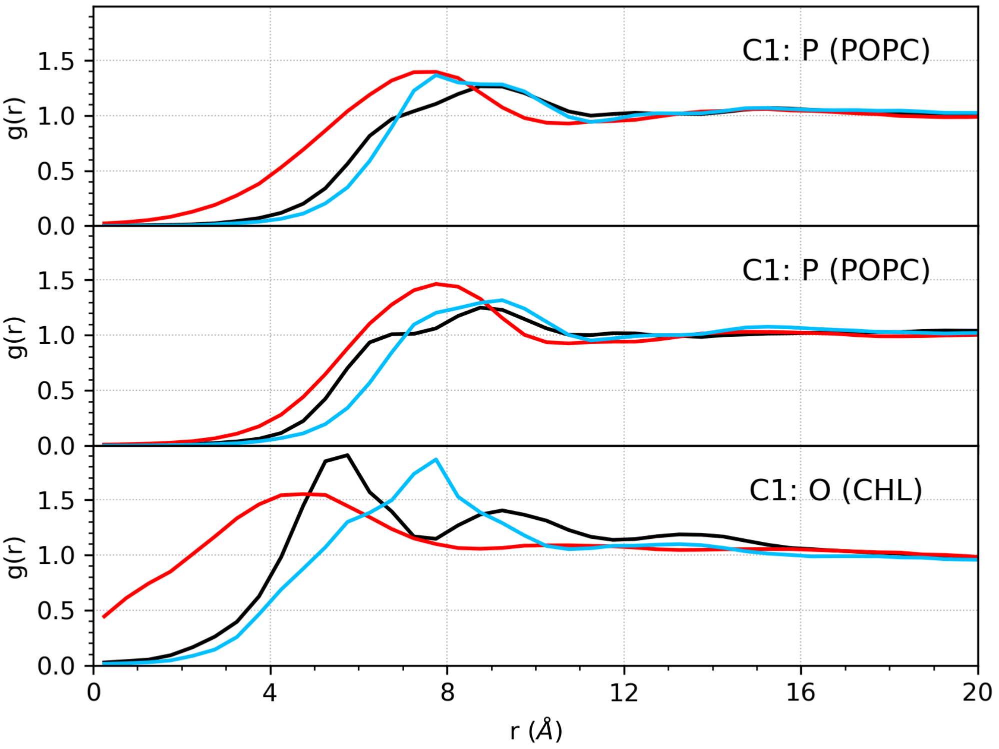

Figure S2: 2D radial pair distribution function g(r) of msVF (black), dsVF (red) and isoVF (cyan) from equilibrium MD simulations in POPC (top) and POPC:CHL (middle and bottom) bilayers, respectively. The g(r) calculation was performed between atom C1 of VF dye fluorescein and the phosphorus (P) atom of POPC or the oxygen atom (O) of CHL (see Methods).

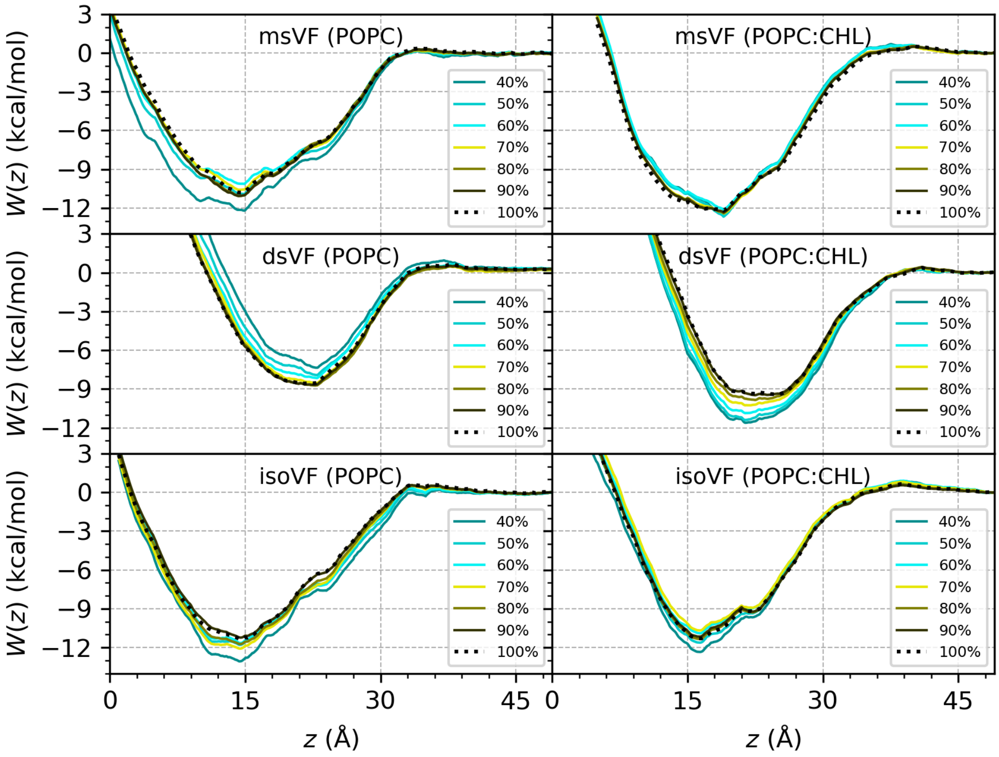

Figure S3: Convergence of the PMF $W(z)$. At a given sampling percentage s, where s shown in the figure ranges from 40% to 100%, $W(z)$ was computed using the first s percentage of the eABF runs, where a positionally, conformationally and orientationally restrained VF dye was transferred reversibly from the lipid membrane to the free solution.

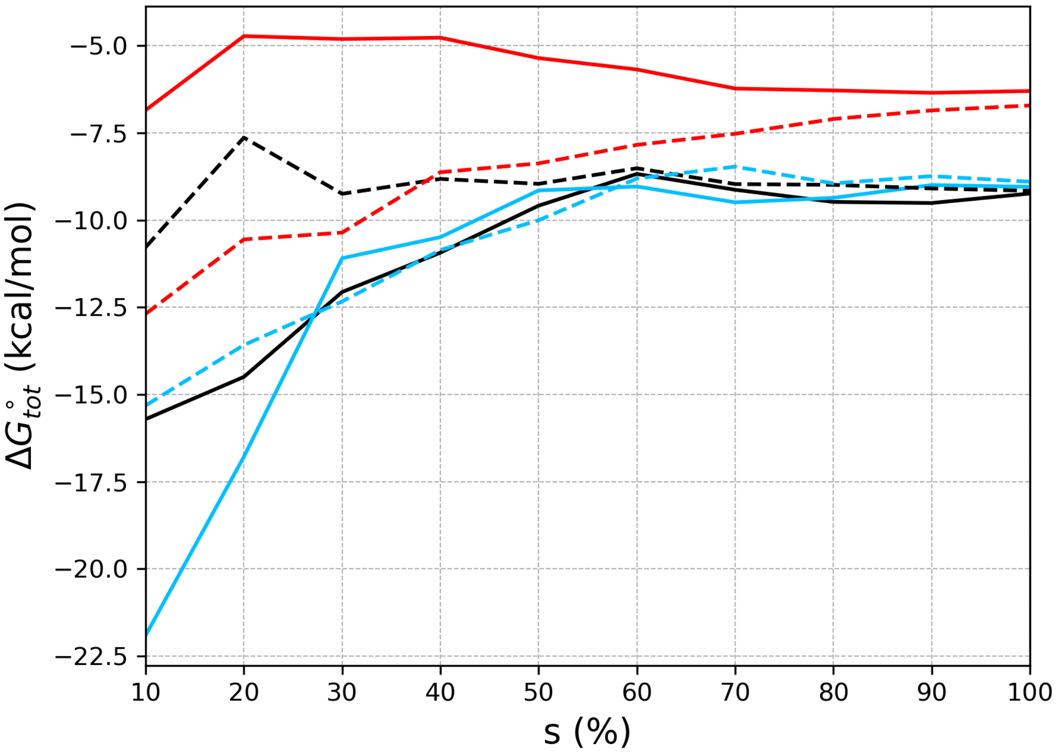

Figure S4: Convergence of $\Delta G_{\rm tot}$ for msVF (black), dsVF (red) and isoVF (cyan) in POPC (solid) and POPC:CHL (dashed) bilayers. At a given sampling percentage s, all components of $\Delta G_{\rm tot}$, except for $\Delta G_{\rm p}^{\rm bound}$ and $\Delta G_{\rm o}^{\rm free}$, were computed using the first s percentage of the corresponding eABF runs, which were then combined to yield $\Delta G_{\rm tot}$ via Eq. 4.

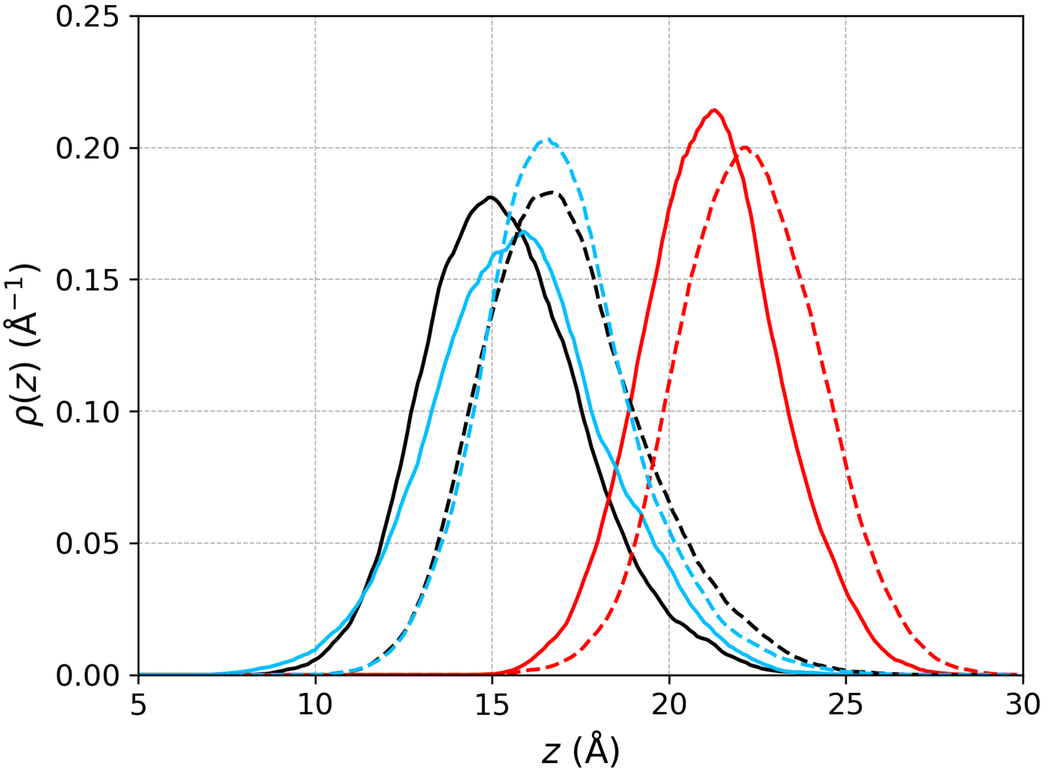

Figure S5: Probability density distribution of the center-of-mass z-coordinate of msVF (black), dsVF (red) and isoVF (cyan) from equilibrium MD simulations in POPC (solid) and POPC:CHL (dashed) bilayers, respectively.

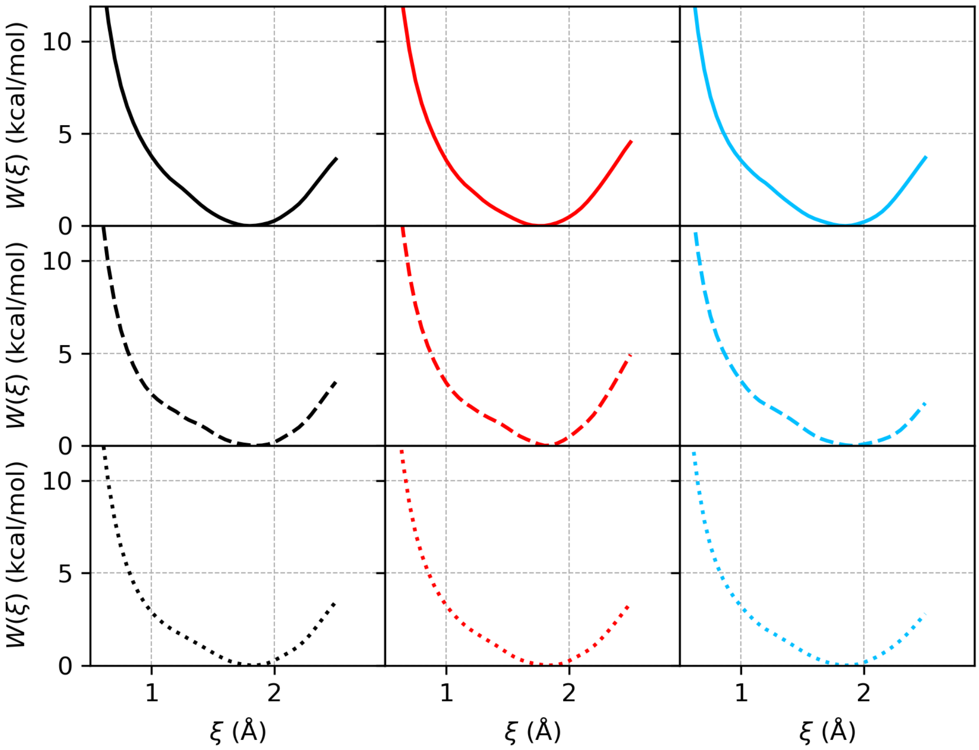

Figure S6: $W(\xi)$ for msVF (black), dsVF (red) and isoVF (cyan) in POPC (solid) and POPC:CHL (dashed) bilayers, as well as in water (dotted).

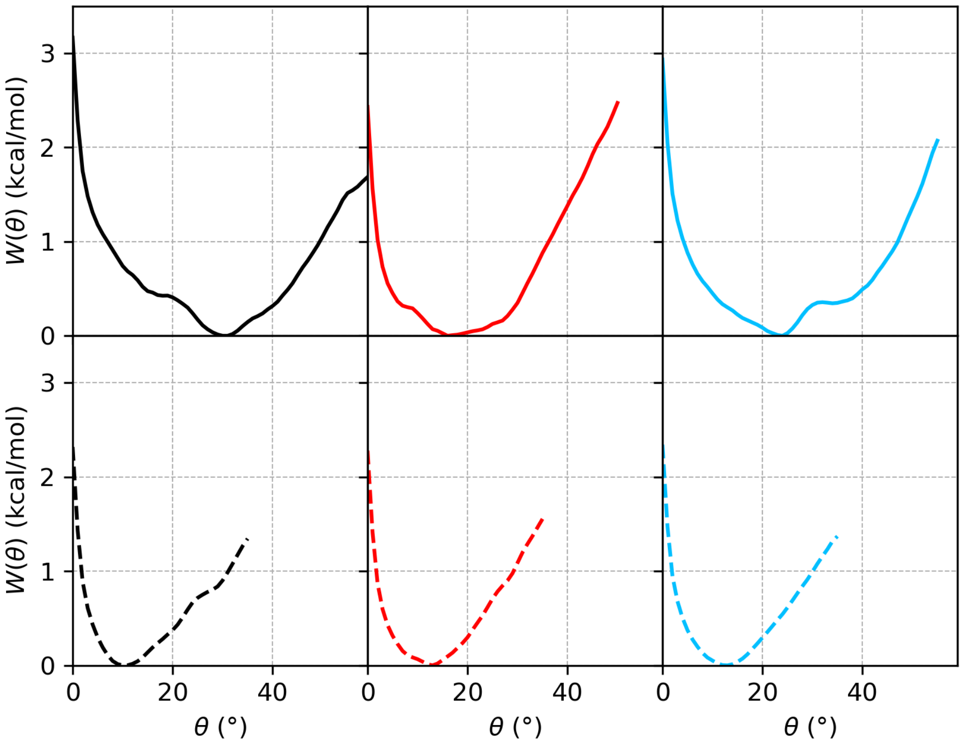

Figure S7: Orientational PMF $W(\theta)$ for msVF (black), dsVF (red) and isoVF (cyan) in POPC (solid) and POPC:CHL (dashed) bilayers.
